# Supplementary material for: Magnons from time-dependent density-functional perturbation theory and nonempirical Hubbard functionals
Source: NPJ Comput Mater. 2025 Apr 16;11(1):100. doi: 10.1038/s41524-025-01570-0 (PMC12003185; doi:10.1038/s41524-025-01570-0)
Supplement: Supplementary file 1 — Supplementary Material [file 41524_2025_1570_MOESM1_ESM.pdf]

Supplemental information for “Magnons from time-dependent density-functional perturbation theory and nonempirical Hubbard functionals”

Luca Binci*[1,†], Nicola Marzari[1,4], and Iurii Timrov[4,+]

[1]Theory and Simulation of Materials (THEOS), and National Centre for Computational Design and Discovery of Novel Materials (MARVEL), École Polytechnique Fédérale de Lausanne, CH-1015 Lausanne, Switzerland
[4]PSI Center for Scientific Computing, Theory, and Data, 5232 Villigen PSI, Switzerland
[†]Email: lbinci@berkeley.edu
[+]Email: iurii.timrov@psi.ch

S1 Crystal and electronic structure properties of NiO and MnO computed using different functionals

	Method	U (eV)	a (Å)	ϑ (deg)	$\|\boldsymbol{m}\|$ (μ_{B})	E_g (eV)
NiO	LSDA+U	6.26	5.03	33.63	1.60	3.04
	PBE+U	5.42	5.19	33.60	1.66	2.87
	PBEsol+U	5.77	5.10	33.62	1.64	3.12
	Expt.		5.11[a]	33.56[a]	1.77[c]	4.0[e]
					1.90[d]	4.3[f]
MnO	LSDA+U	4.29	5.32	34.16	4.19	1.93
	PBE+U	3.85	5.46	34.12	4.28	1.64
	PBEsol+U	4.05	5.38	34.19	4.23	1.70
	Expt.		5.44[b]	33.56[b]	4.79[c]	4.1[e]
					4.58[d]	3.9±0.4[g]

Table S1. Crystal and electronic structure properties of NiO and MnO as computed using LSDA+U, PBE+U, and PBEsol+U (all spin-polarized) with their respective self-consistent Hubbard U parameters for Ni-3d and Mn-3d states calculated using DFPT, and as measured in experiments. The equilibrium rhombohedral lattice parameter (a), rhombohedral angle (ϑ), magnetic moment ($|\boldsymbol{m}|$), and band gap (E_g) are presented. The experimental values for a and ϑ are determined from the cubic lattice using the experimental lattice parameter (4.17 and 4.43 Å for NiO[a] and MnO,[b] respectively), since experimentally the rhombohedral distortion is not quantified. The angle $\vartheta = 33.56°$ corresponds to the case with no rhombohedral distortion. Ref.[a]: 1, Ref.[b]: 2, Ref.[c]: 3, Ref.[d]: 4, Ref.[e]: 5, Ref.[f]: 6, Ref.[g]: 7.

*Present address: Department of Materials Science & Engineering, University of California Berkeley, Berkeley, CA, 94720, USA; Materials Sciences Division, Lawrence Berkeley National Laboratory, Berkeley, CA, 94720, USA

S2 Behavior of Lanczos coefficients

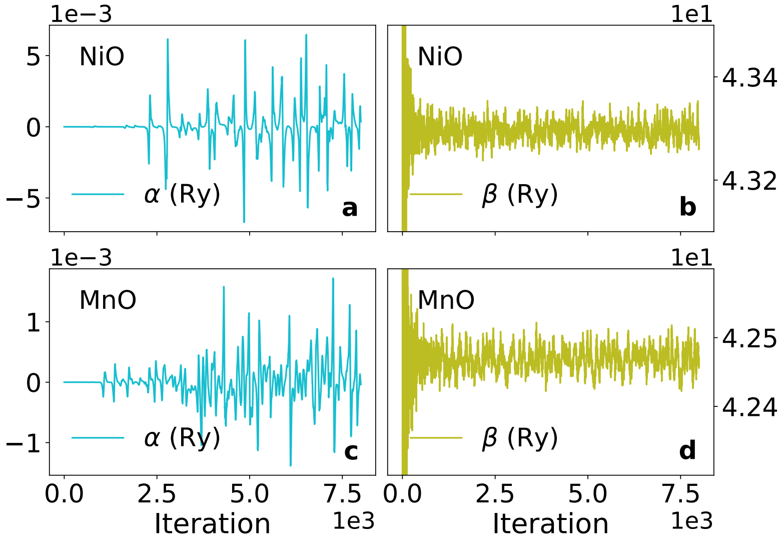

Figure S1. Behavior of the average even/odd Lanczos coefficients α_n (panels **a** and **c**) and β_n (panels **b** and **d**) as a function of the number of Lanczos iterations n within TDDFPT+U for NiO and MnO.

S3 The $q \to 0$ limit of the magnon dispersion

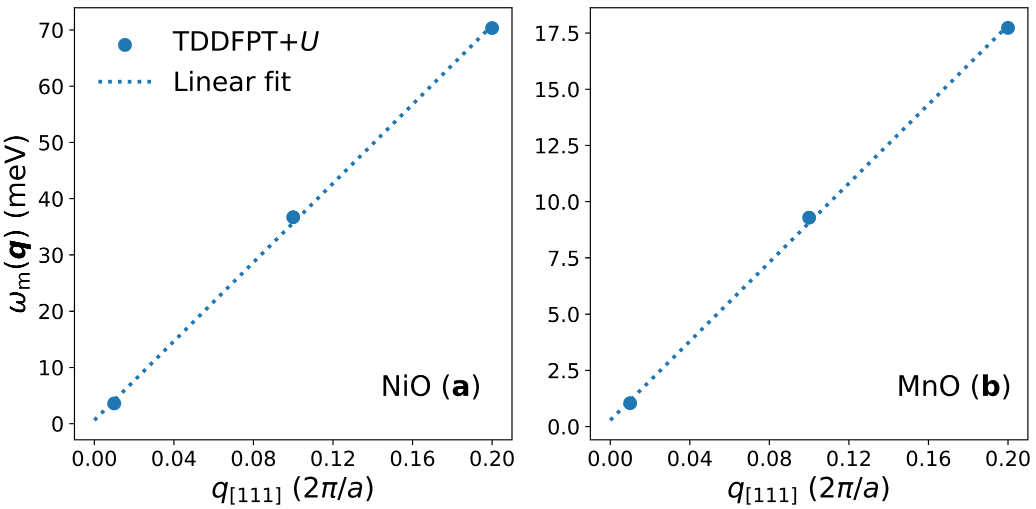

Figure S2. Magnon dispersion for NiO (a) and MnO (b) for small transferred momenta $\boldsymbol{q}$ along the [111] direction (Γ-X) and a linear fit.

References

1. Schmahl, N., Barthel, J. & Eikerling, G. *Z. Anorg. Allg. Chem.* **332**, 230, DOI: 10.1002/zaac.19643320503 (1964).
2. Sasaki, S., Fujino, K., Taki-Uchi, Y. & Sadanag, R. *Acta Cryst. A* **36**, 904, DOI: 10.1107/S0567739480001908 (1980).
3. Fender, B., Jacobson, A. & Wedgwood, F. *J. Chem. Phys.* **48**, 990, DOI: 10.1063/1.1668855 (1968).
4. Cheetham, A. & Hope, D. *Phys. Rev. B* **27**, 6964, DOI: 10.1103/PhysRevB.27.6964 (1983).

5. Kurmaev, E. *et al.* *Phys. Rev. B* **77**, 165127, DOI: 10.1103/PhysRevB.77.165127 (2008).
6. Sawatzky, G. & Allen, J. *Phys. Rev. Lett.* **53**, 2339, DOI: 10.1103/PhysRevLett.53.2339 (1984).
7. van Elp, J., Potze, R., Eskes, H., Berger, R. & Sawatzky, G. *Phys. Rev. B* **44**, 1530, DOI: 10.1103/PhysRevB.44.1530 (1991).
